# Editorial: Revisiting the Effectiveness of Transcranial Direct Current Brain Stimulation for Cognition: Evidence, Challenges, and Open Questions

**DOI:** 10.3389/fnhum.2017.00448

**Published:** 2017-09-08

**Authors:** Evangelia G. Chrysikou, Marian E. Berryhill, Marom Bikson, H. Branch Coslett

**Affiliations:** ^1^Department of Psychology, University of Kansas Lawrence, KS, United States; ^2^Department of Psychology, University of Nevada Reno, NV, United States; ^3^Department of Biomedical Engineering, City University of New York New York, NY, United States; ^4^Department of Neurology, University of Pennsylvania Philadelphia, PA, United States

**Keywords:** transcranial direct current stimulation (tDCS), neuroenhancement (NE), noninvasive brain stimulation, cognition and emotion, neurorehabilitation

Over the past 15 years, there has been an explosion of interest in the use of noninvasive brain stimulation approaches to study the brain. Some studies have suggested that transcranial Direct Current Stimulation (tDCS) in particular can elicit positive effects on performance for many aspects of cognition, including working memory, attention, executive function, language, and numerical competence. A growing literature further indicates that tDCS can provide potentially long-lasting benefits for patient rehabilitation, ameliorating wide-ranging conditions such as aphasia, pain, major depression, tinnitus, and migraine, among others.

It is well-accepted that tDCS is a well-tolerated, noninvasive technique that involves the application of low levels of direct current (1–2 mA, 10–30 min) through electrodes placed on the scalp to alter the neural activity of underlying neural populations. It is often assumed that during and immediately after application, cortical excitability increases under the anode electrode because of neuron soma depolarization, whereas cortical excitability decreases under the cathode electrode because of neuron soma hyperpolarization (Purpura and McMurtry, 1965; Nitsche and Paulus, 2000, 2001)—however, both the outcomes and mechanisms of tDCS are more complex (Giordano et al., 2017; Jamil et al., 2017; Kronberg et al., 2017). Long-term effects of tDCS have been linked to neuroplasticity following LTP-like changes in synaptic strength between stimulated neurons involved in task performance (Reato et al., 2010; Rahman et al., 2015). An advantage of the procedure relative to other brain stimulation techniques is its reliable sham manipulation: active tDCS is silent, does not induce muscle twitches, and it is not immediately distinguishable from sham stimulation, thus allowing for double blinded studies (but see Giordano et al., 2017). Critically, the existing availability of devices that can administer tDCS, its ease of use, and its excellent safety profile underscore the potential of tDCS as a tool for improving cognitive performance in healthy populations, stabilizing cognition in those who are at high risk for cognitive decline, and providing adjuvant therapy for those in need of cognitive rehabilitation.

Nevertheless, despite these recent advances in the use of the procedure, the precise neurobiological mechanisms underlying tDCS effects in humans remain insufficiently understood. Tempering the enthusiasm for this methodology, a number of recent quantitative reviews of both neurophysiological and cognitive studies using tDCS have raised questions regarding its effectiveness to induce reliable neuroplastic changes that measurably affect cognition in neurotypical or patient populations (Horvath et al., 2014, 2015a,b). Additional concerns pertain to the replicability of the findings reported in the existing literature and the specification of the precise conditions under which positive tDCS effects can be obtained (Mancuso et al., 2016). Overall, there is a great deal of variability in the robustness of tDCS-linked cognitive outcomes that may be largely attributed to small and heterogeneous sample sizes, the scarcity of data on dose-response effects, and substantial methodological diversity across laboratories. These limitations are exacerbated by the aforementioned lack of understanding of the precise mechanistic effects of a given tDCS protocol on the brain over short- and long-timeframes and in the context of particular tasks.

As researchers using tDCS, we are acutely aware of the limitations in interpretation and application imposed by these gaps in knowledge about the procedure and we are highly motivated to fill them. To satisfy this goal, this Frontiers Research Topic brings together 11 articles from leading experts in the field of non-invasive brain stimulation that aim to address several of the above-mentioned questions associated with tDCS, as well as examine the strength of the evidence regarding the potential of tDCS to modulate different aspects of cognition.

The resulting collection of articles is divided into two clusters centered around (a) methodological issues and perspectives and (b) the influence of individual and group differences in guiding tDCS effects. The first part of the E-book begins with a review by Esmaeilpour et al. of the outcomes of human trials using tDCS in psychiatric populations that helps situate the current literature regarding effectiveness of experimental designs and tDCS stimulation protocols. Harty et al. provide a strong rationale for use of mediation and moderation analyses when examining interactions between tDCS interventions, neural dynamics, and behavior. In turn, Minarik et al. focus on the importance of appropriate sample sizes to ensure replicability of tDCS findings, while highlighting the likelihood of overestimating effect sizes based on the published literature to date. Two additional papers in this section examine the effects of current polarity, intensity, and stimulation site for tDCS effects. Karuza et al. examine the consequences of parametric variations in current polarity and stimulation intensity for a cognitive control task, whereas Weinberger et al. review how interactions among task demands, tDCS polarity, and stimulation site can measurably enhance flexible thinking.

The second group of papers takes a sharp look at several individual and group differences factors that can determine the strength of tDCS effects, consideration of which is required to advance the interpretability of tDCS research. Wiegand et al. point out the essential role of genetically-determined variations in neural activity in predicting tDCS outcomes, which is particularly notable in studies of executive function. Likewise, Hsu et al. show that baseline differences in working memory performance interact with task difficulty and other state-dependent individual differences factors to determine responsiveness to tDCS. Rosen et al. similarly show that individual level of expertise determines whether anodal tDCS will enhance or impede performance in a jazz improvisation task. The last three papers highlight the importance of such individual and group differences factors for tDCS outcomes in clinical settings. Two empirical papers, one by McConathey et al. and a second by Norise et al., demonstrate how baseline measures of patient severity and task specificity can determine the efficacy of tDCS for the treatment of aphasia. Lastly, Mervis et al. review the extent of anodal or cathodal tDCS-guided improvements for different aspects of cognition in schizophrenia.

We are pleased with the breadth of topics covered in this collection and the issues addressed. Yet it is clear that each article raises a series of new questions in need of answers that will require much future research. For this goal to be achieved, it is critical to develop appropriate statistical methods and power analyses that will allow sufficient consideration of complex interactions among an extensive set of factors shown to drastically influence tDCS outcomes. Additionally, substantial work on the neurobiological mechanisms associated with tDCS effects in the brain is acutely needed. We hope this compilation will serve as a starting point for these investigations by framing the challenges and future directions for the use of tDCS that can determine its potential as a reliable method for cognitive rehabilitation, maintenance, or enhancement.

## Author contributions

All authors listed have made a substantial, direct and intellectual contribution to the work, and approved it for publication.

### Conflict of interest statement

The City University of New York has patents on brain stimulation with MB as inventor. MB has equity in Soterix Medical Inc. The other authors declare that the research was conducted in the absence of any commercial or financial relationships that could be construed as a potential conflict of interest.
